# Metabolic Disturbances in the Striatum and Substantia Nigra in the Onset and Progression of MPTP-Induced Parkinsonism Model

**DOI:** 10.3389/fnins.2018.00090

**Published:** 2018-02-20

**Authors:** Yi Lu, Xiaoxia Zhang, Liangcai Zhao, Changwei Yang, Linlin Pan, Chen Li, Kun Liu, Guanghui Bai, Hongchang Gao, Zhihan Yan

**Affiliations:** ^1^Department of Radiology, The Second Affiliated Hospital and Yuying Children's Hospital of Wenzhou Medical University, Wenzhou, China; ^2^Institute of Metabonomics & Medical NMR, School of Pharmaceutical Sciences, Wenzhou Medical University, Wenzhou, China

**Keywords:** Parkinson's disease, ^1^H NMR, striatum, metabolism, neurotransmitter

## Abstract

Metabolic confusion has been linked to the pathogenesis of Parkinson's disease (PD), while the dynamic changes associated with the onset and progression of PD remain unclear. Herein, dynamic changes in metabolites were detected from the initiation to the development of 1-Methyl-4-phenyl-1,2,3,6-tetrahydropyridine (MPTP) -induced Parkinsonism model to elucidate its potential metabolic mechanism. *Ex vivo* ^1^H nuclear magnetic resonance (NMR) spectroscopy was used to measure metabolite changes in the striatum and substantia nigra (SN) of mice at 1, 7, and 21 days after injection of MPTP. Metabolomic analysis revealed a clear separation of the overall metabolites between PD and control mice at different time points. Glutamate (Glu) in the striatum was significantly elevated at induction PD day 1 mice, which persisted to day 21. N-acetylaspartate (NAA) increased in the striatum of induction PD mice on days 1 and 7, but no significant difference was found in striatum on day 21. Myo-Inositol (mI) and taurine (Tau) were also disturbed in the striatum in induction PD day 1 mice. Additionally, key enzymes in the glutamate-glutamine cycle were significantly increased in PD mice. These findings suggest that neuron loss and motor function impairment in induction PD mice may be linked to overactive glutamate-glutamine cycle and altered membrane metabolism.

## Introduction

Parkinson's disease (PD) is one of the most common neurodegenerative diseases found in the aging population. As the worldwide population ages and life expectancy increases, the number of people with PD is expected to rise by more than 50% by 2030 (Kalia and Lang, 2015). The attendant motor disorders, such as resting tremor, rigidity, bradykinesia or slowness, gait disturbance, and postural instability, significantly decrease the quality of life (Fasano et al., 2012). As individuals have an increased life span, increased PD and its treatment can lead to a severe burden to patients, caregivers, and social health institutions (Nagy et al., 2012).

A metabolic abnormality was previously implicated in the pathogenesis of PD. Dopamine (DA) and its metabolites, 3, 4-dihydroxyphenylacetic (DOPAC), and homovanillic acid (HVA), are critical in the physiopathology of PD with motor dyskinesia and were used to validate the pharmacology of L-Dopa and other therapies (Smith et al., 2014). Tryptophan, serotonin, and the serotonin metabolite 5-HIA were also thought to be related to the psychiatric symptoms of PD (Hatano et al., 2016). However, dopamine modulators can cause serious side effects and often lose effectiveness. The discovery of other metabolites that are altered in the pathogenesis of the neurodegeneration may be involved in the underlying molecular pathways of PD, such as oxidative stress, inflammation, and glial reactions (Dawson and Dawson, 2003; Niranjan, 2014).

Wen et al. (2015) found in a PD rat model with 6-hydroxydopamine (6-OHDA) lesions showed significantly decreased acetylcholine (ACh) and moderately decreased noradrenaline (NA) concentrations in the ventrolateral thalamic nucleus. Ma et al. (2015) revealed that regional glucose metabolism changed in parkinsonism non-human primates by FDG PET. Alterations in the bilirubin-to-biliverdin ratio and ergothioneine in the serum indicate altered oxidative stress intensity, suggesting elevated oxidative stress and/or insufficient ability for scavenging free radicals, as was shown in the work of Hatano et al. (2016). Therefore, studying metabolic changes in cerebral metabolites at the molecular level could uncover novel pathophysiologic mechanisms involved in PD.

Proton nuclear magnetic resonance (^1^H NMR)-based metabolomics, a powerful approach to study the brain energy metabolism and neurotransmission, has been widely used in psychosis (Chitty et al., 2015), brain tumor (Zhang et al., 2014), and other cerebral diseases. Compared to other metabolomics analytical techniques, NMR has some unique advantages, such as minimal sample processing, robust reproducibility, and high throughput (Lei and Powers, 2013). Using this approach, our previous work showed disturbances of glutamate (Glu), glutamine (Gln), and γ-Aminobutyric acid (GABA) in the Gln-Glu-GABA cycle (GGC) in the striatum of PD rats (Zheng et al., 2015). Although the metabolic abnormality had been previously found to be involved in the pathogenesis of PD, dynamic changes associated with the onset and progression of PD remain to be investigated.

To comprehensively profile changes in metabolites associated with the onset and progression of PD, we used 1-Methyl-4-phenyl-1,2,3,6-tetrahydropyridine (MPTP)-induced PD mice, the most widely accepted dopaminergic neurotoxin used in rodents for studying the progression and mechanisms involved in PD (Jackson-Lewis and Przedborski, 2007). With the goals of exploring the cerebral metabolic bases of the occurrence and development of PD, NMR-based metabolomics was performed to study the kinetic changes in metabolites in the striatum and substantia nigra (SN) of MPTP-induced PD mice.

## Results

### TH neuronal contents and behavioral performance

**Figure 1:**
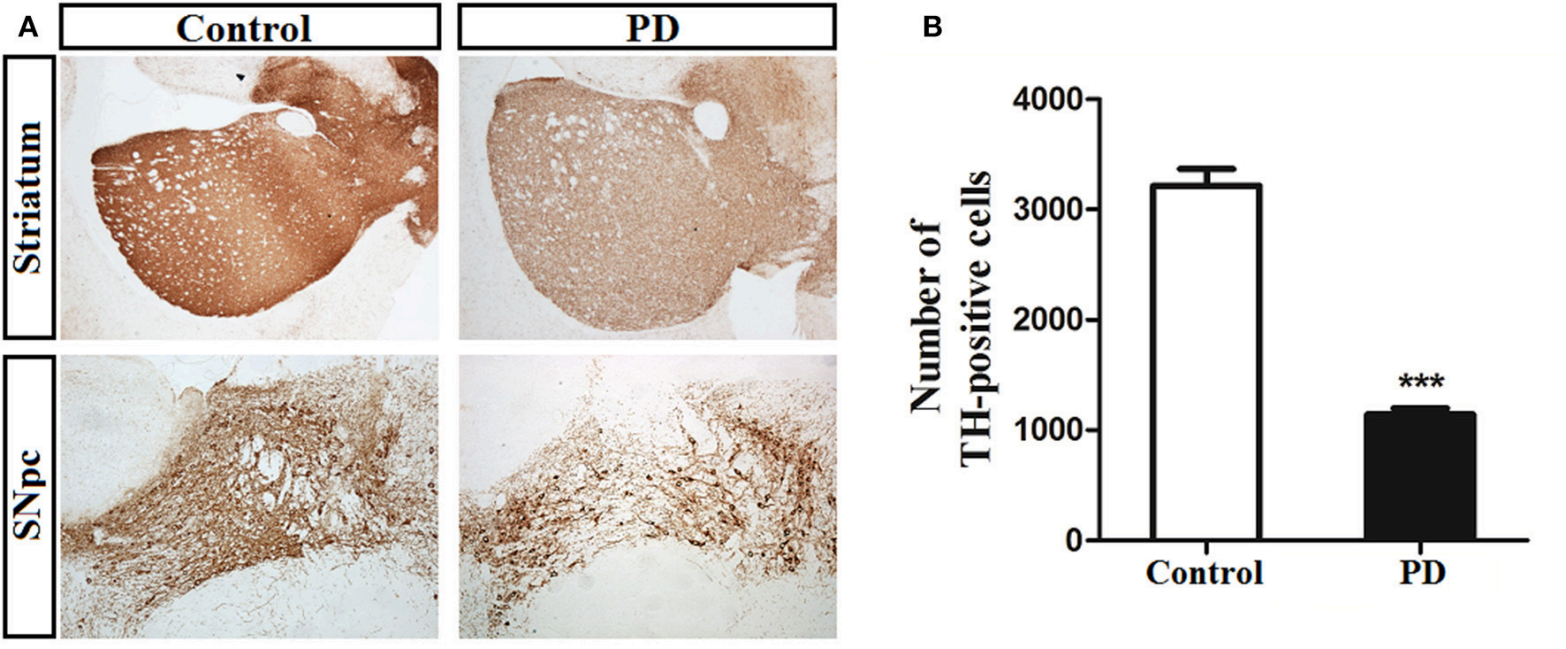
Immunohistochemical staining of tyrosine hydroxylase (TH) in the substantia nigra pars compacta (SNpc) and striatum. **(A)** Illustration of toxicity of MPTP on TH in both SNpc and striatum at the first day after the last treatment. **(B)** MPTP administration induced a significant decrease (64%) number of TH-positive neurons in SNpc of PD group compared with control group (3,213 ± 348 vs. 1,142 ± 122). Bar represented means ± *SD* of three mice per group (^***^p < 0.001).

Tyrosine hydroxylase (TH) can be used to label dopaminergic afferents and is commonly used to verify the success of a PD model (Jackson-Lewis and Przedborski, 2007). Microscopic examinations identified representative TH-positive neurons in the SN and striatum from control and induction PD day 1 mice (Figure 1A). MPTP administration led to a significant reduction in the number of TH-positive cells in the SN compared with the control group (1,142 ± 122 vs. 3,213 ± 348, *p* < 0.001), demonstrating the successful establishment of the PD model (Figure 1B).

**Figure 2:**
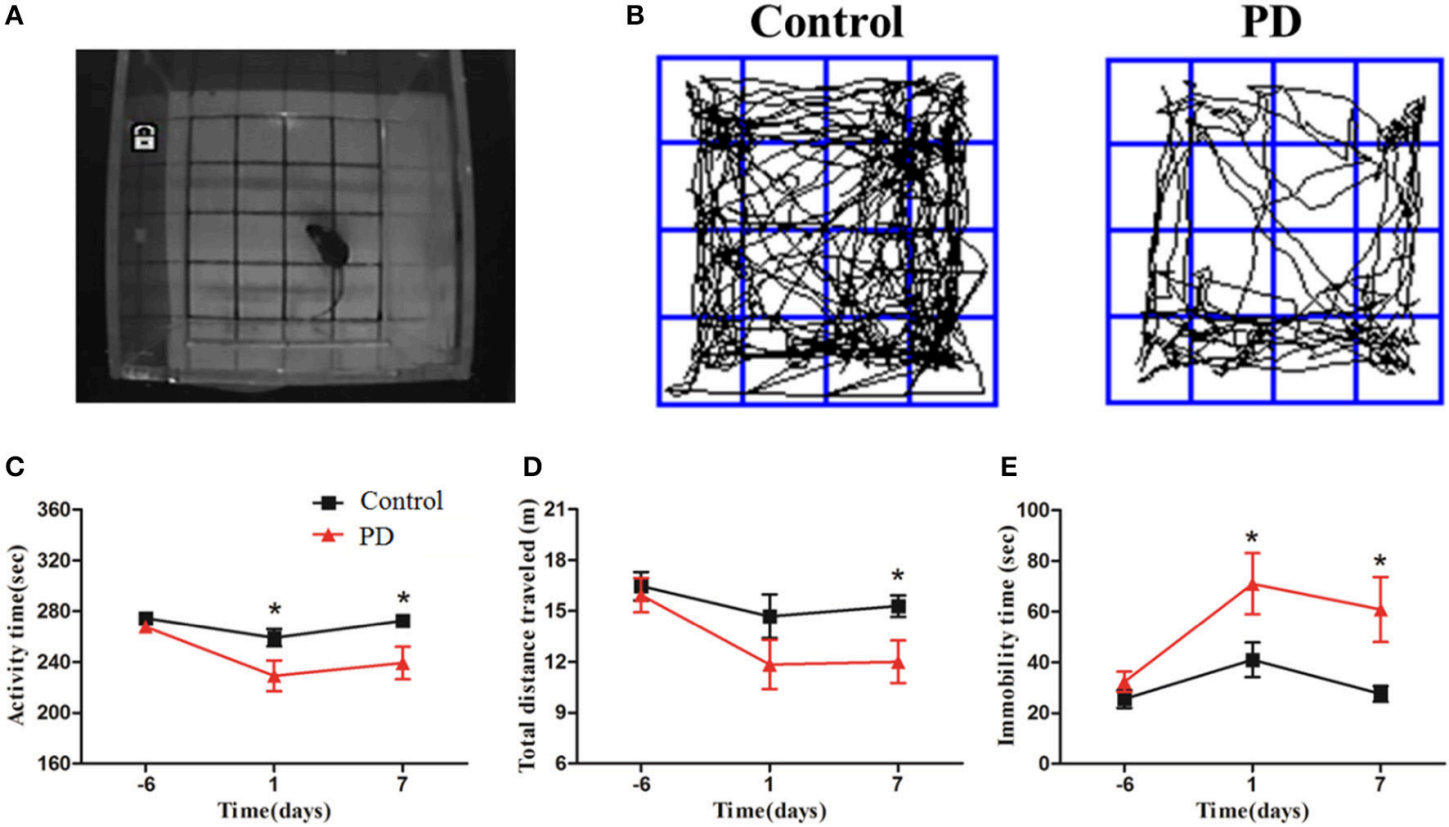
MPTP deteriorated Behavioral alteration of mice in the open field test. **(A)** The open field test was conducted in the chamber measuring 25 × 25 × 45 cm with an automated video tracking system. **(B)** Representative examples of movement path was selected at 1 day after last treatment. The activity time, total distance traveled and immobility time **(C–E)** were recorded and analyzed at 3 time points (6 days before the last treatment, 1 and 7 days after the last treatment). All data were shown as means ± SEM, ^*^p < 0.05.

Mice were subjected to an open field test 1 day before modeling, and then 1 and 7 days after the injection of MPTP or normal saline (Figure 2A). Significant reductions in the movement path, activity time, and total distance were observed in the PD group at different time points (Figures 2B–D). In addition, the immobility time in the PD group was significantly increased compared to control mice (Figure 2E). These behavioral findings are consistent with the formation of lesions and motor symptoms in MPTP-induced Parkinsonism mice model.

### NMR-based metabolic profiling of the striatum of mice

**Figure 3:**
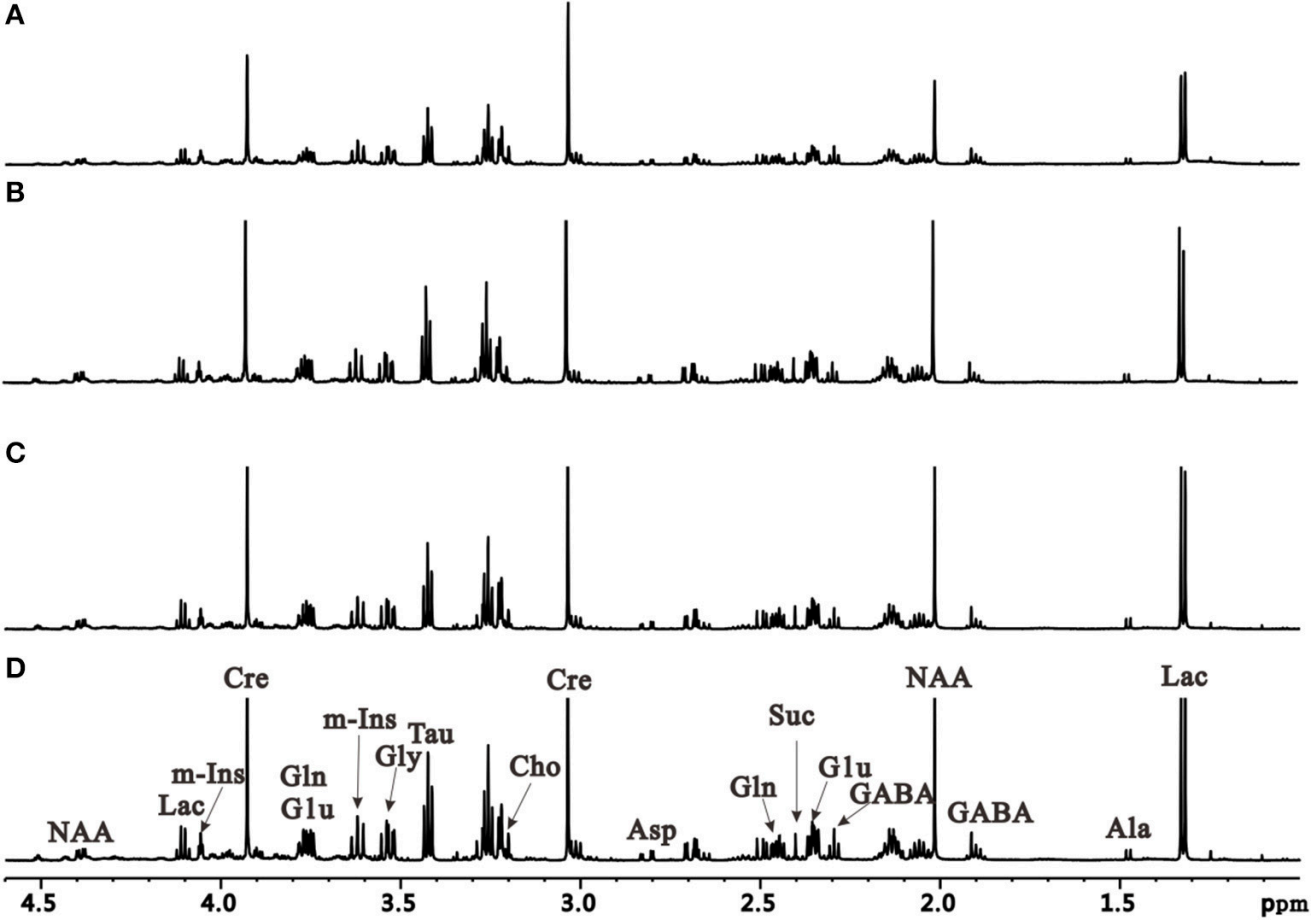
Representative 1H NMR spectra of the right striatum extracts obtained from the control group at the first day after administration **(A)** and induction PD group, respectively at 1 day **(B)**, 7 days **(C)**, and 21 days **(D)**.

To further characterize dynamic changes of metabolites that occur during PD progression, we performed an unbiased metabolic analysis of striatum and SN tissues from control and induction PD mice at different time points. Representative ^1^H NMR spectra of striatum extracts obtained from control and induction PD mice are shown in Figure 3. Assignments presented in Figure 3D were based on our previously published work (Gao et al., 2007) using the 600 MHz library of Chenomx NMR suite 7.0 (Chenomx Inc., Edmonton, Canada). The ^1^H NMR spectra of brain tissue extracts allows for the simultaneous measurement of the numbers of endogenous metabolites, including Glu, GABA, Gln, lactate (Lac), alanine (Ala), N-acetyl aspartate (NAA), succinate (Suc), aspartate (Asp), creatine (Cre), choline (Cho), taurine (Tau), glycine (Gly), and myo-inostitol (mI).

**Figure 4:**
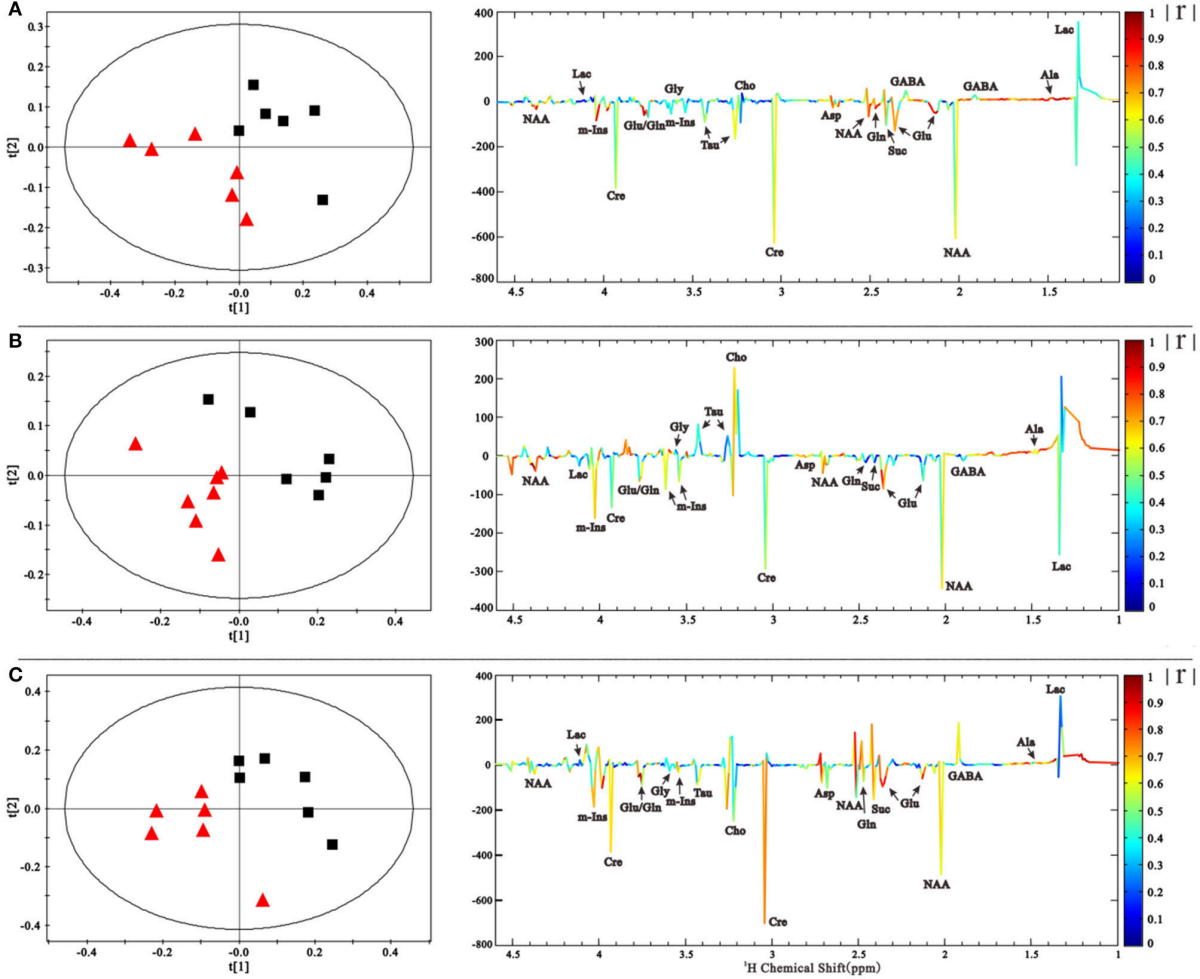
PLS-DA scores plot based on the ^1^H NMR spectra of the right striatum samples showed that PD group (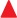) is clearly separated from control group (■) at 1, 7, and 21 days **(A–C)**. The coefficient-coded loading plots corresponding to PLS-DA revealed the metabolites with large intensities responsible for the discrimination of the corresponding score plots.

NMR spectra were normalized to the internal standard TSP concentration to evaluate the relative content of metabolites and binned to reduce data dimensions for further metabolomic analyses. We conducted a Partial Least Squares-Discriminant Analysis (PLS-DA), a method that incorporates elements from principal component analysis, regression, and linear discriminant analysis, which revealed a clear separation of the overall metabolite levels in the right striatum between induction PD mice and control mice on days 1, 7, and 21 (Figure 4). Further evaluation of the metabolome using a corresponding loading plot, shown with color-coded correlation coefficients (|r|) of metabolites, revealed changed levels of Glu, Gln, NAA, Asp, mI, Cre, and Ala, indicating that neurotransmitter regulation and other metabolites disturbances could be involved in the progression of PD.

### Metabolite changes in various brain regions

**Figure 5:**
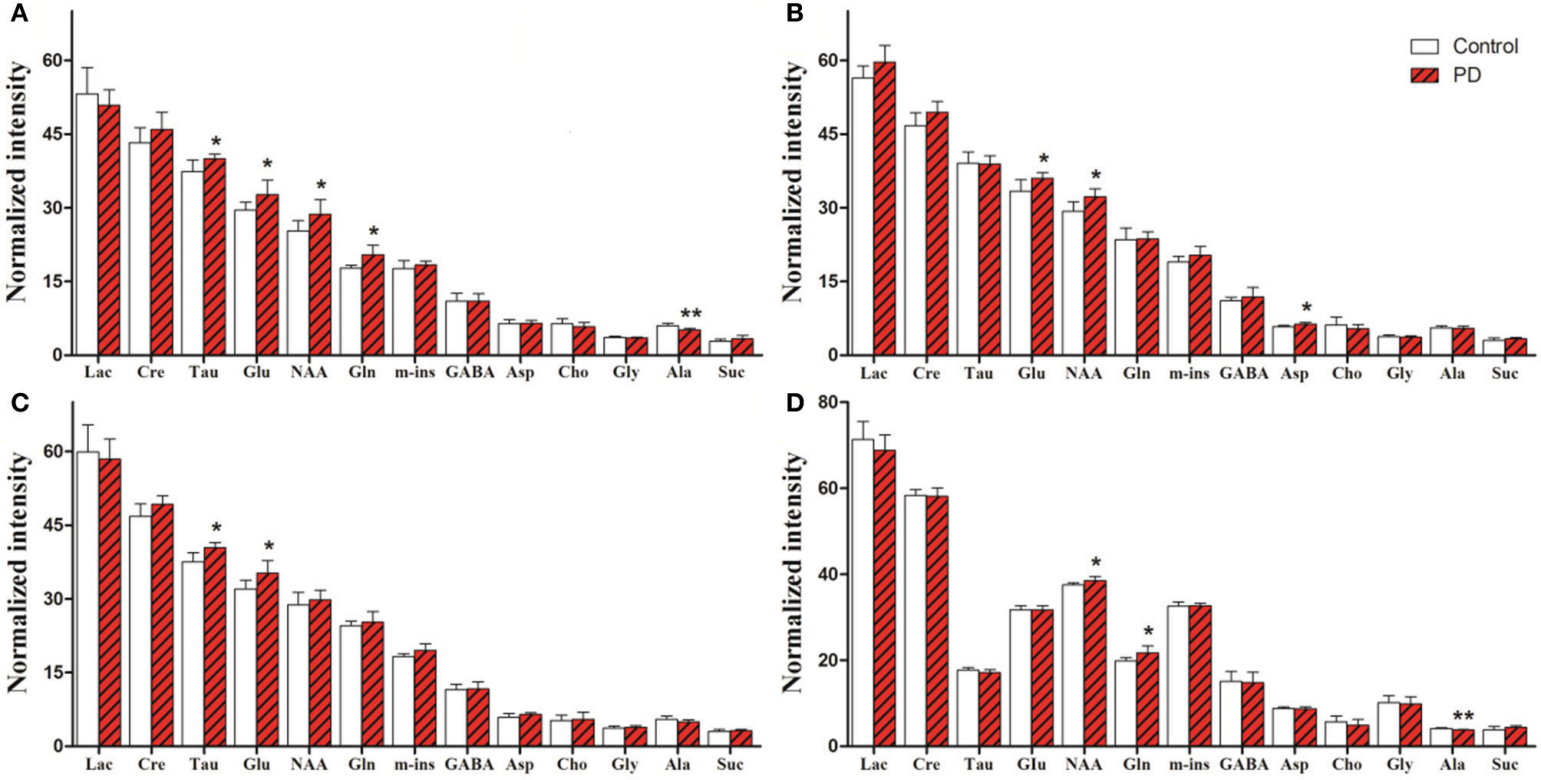
Metabolic alterations obtained from the striatum of induction PD day 1, 7, 21 group **(A–C)** and the substantia nigra of induction PD day 1 group **(D)**, respectively. The results are expressed as mean ± *SD*, ^*^p < 0.05, ^**^p < 0.01.

For univariate statistical analysis, relative concentrations of specific metabolites were calculated for each subject by subtracting the integrals of the signals. Levels of all identified metabolites in the right striatum and SN are shown in Figure 5. In the right striatum of induction PD mice at day 1 (Figure 5A), compared with the control group we found higher levels of neurotransmitters, including Glu (32.72 ± 2.90 vs. 29.55 ± 1.59, *p* = 0.041) and Gln (20.46 ± 1.97 vs. 17.78 ± 0.53, *p* = 0.009), the neuronal marker NAA (28.73 ± 2.93 vs. 25.30 ± 2.07, *p* = 0.041) and the antioxidant Tau (39.99 ± 0.91 vs. 37.39 ± 2.30, *p* = 0.027). By contrast, levels of the energy-related metabolite Ala decreased (5.16 ± 0.32 vs. 6.03 ± 0.51, *p* = 0.006). In the induction PD day 7 group (Figure 5B), metabolic profiles revealed higher levels of NAA (32.23 ± 1.64 vs. 29.34 ± 1.91, *p* = 0.014), Glu (36.00 ± 1.15 vs. 33.38 ± 2.34, *p* = 0.024), and Asp (6.32 ± 0.40 vs. 5.86 ± 0.23, *p* = 0.033). In addition, higher levels of Glu (35.25 ± 2.54 vs. 31.98 ± 1.84, *p* = 0.029) and Tau (40.42 ± 1.08 vs. 37.53 ± 1.86, *p* = 0.008) were detected in the induction PD day 21 group (Figure 5C). In the SN, PD mice were marked by high concentrations of NAA (38.55 ± 0.93 vs. 37.55 ± 0.47, *p* = 0.048) and Gln (21.55 ± 1.53 vs. 19.72 ± 0.80, *p* = 0.031; Figure 5D).

**Figure 6:**
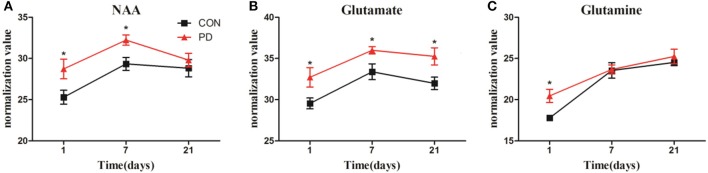
Changes in the levels of NAA **(A)**, Glu **(B)**, and Gln **(C)** obtained from ^1^H NMR spectra of right striatum samples collected from PD and control group of 1, 7, and 21 days after last administration. The results were expressed as means ± SD, ^*^p < 0.05.

Changes in the levels of NAA, Glu, and Gln in the striatum throughout the progression of MPTP-induced Parkinsonism are shown in Figure 6. NAA levels were significantly higher in the induction PD day 1 and 7 groups compared to the control group, while no difference was observed at induction PD day 21. Meanwhile, Glu levels were elevated at day 1, then reached a maximum at day 7, and remained elevated until day 21. In addition, Gln levels were significantly elevated at day 1, but not at days 7 or 21.

### Key enzymes in the glutamate-glutamine cycle

**Figure 7:**
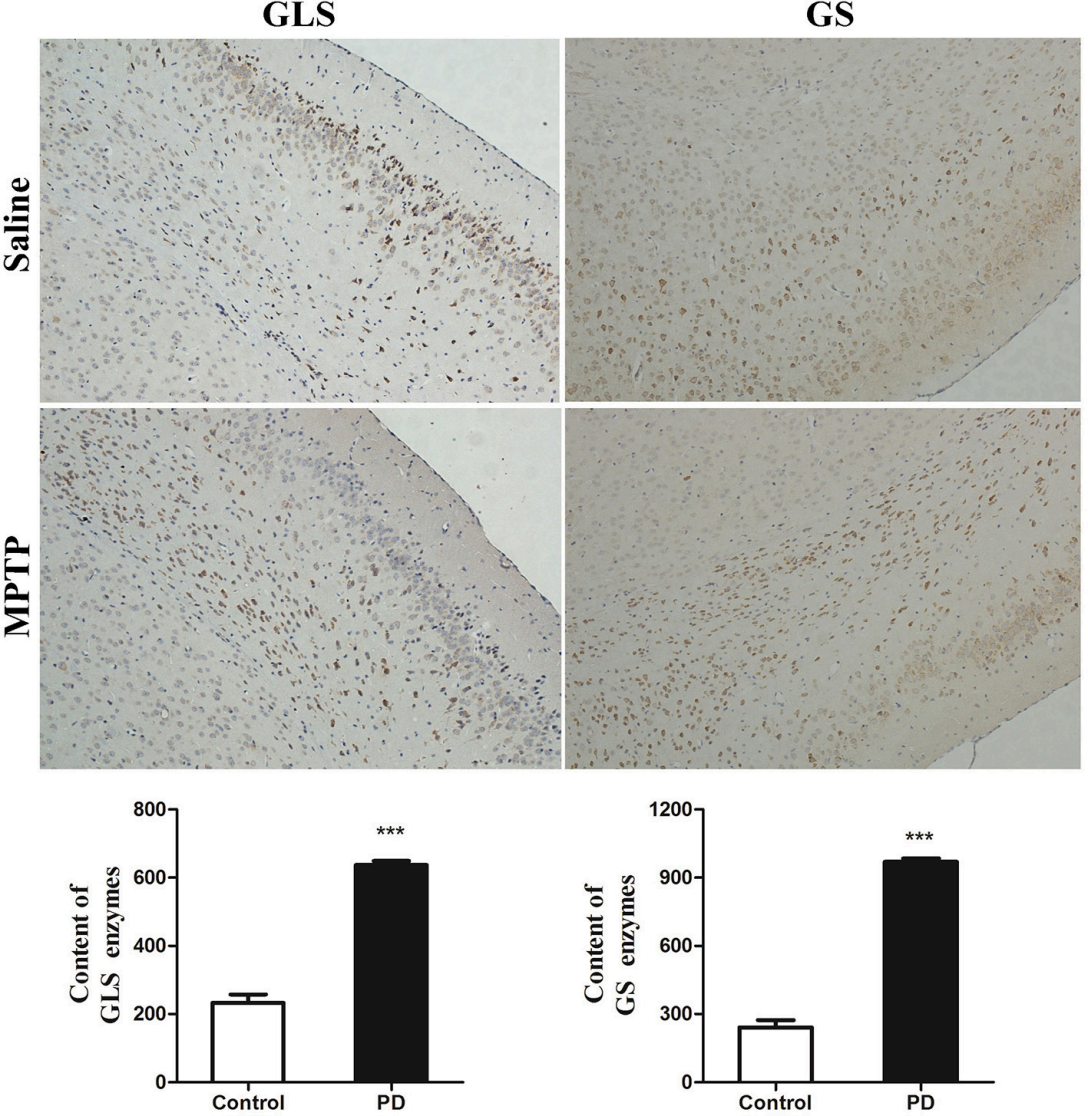
Key enzymes were disturbed by MPTP in the disordered cycle of Glu-Gln-GABA. The deep dyed brown cytoplasm was represented as GLS and GS enzymes, by immunohistochemical staining. Content of GLS and GS enzymes were expressed as means ± SD, ^***^p < 0.001.

The metabolic changes in Glu and Gln in PD mice suggested that the GGC was involved in the progression of MPTP-induced Parkinsonism. To further explore the reasons whereby GGC influenced PD in mice, levels of selected key enzymes involved in this cycle, including GS and GLS, were measured. GS, an ubiquitous enzyme present in the astroglial cytoplasm and involved in the formation of Gln from Glu (Hertz et al., 1999), was shown to be elevated in the subcortex of PD mice (Figure 7). In addition, a similar result was shown in the GLS, which was consistent with the increased conversion of Gln into Glu. Collectively, these findings suggest that an overactive GGC could be observed during the progression of MPTP-induced PD in mice.

## Discussion

PD, a multi-centric neurodegenerative disorder, has been shown to be associated with disturbed systemic metabolism (Bogdanov et al., 2008). However, variations in brain metabolites at different time points during the progression remain to be firmly established. In this present study, we used MPTP-induced PD mice to investigate dynamic changes in metabolite levels using high resolution *ex vivo* ^1^H NMR spectroscopy. Metabolomics analysis showed that multiple time- and region-dependent neurochemical metabolite perturbations occurred, including NAA, Glu, Gln, mI, and Tau in the striatum and SN. These changes could be involved in the initiation and development of PD pathogenesis.

NAA has been widely used as a marker of neuronal density as its concentration is reduced in cases of neuronal loss (Delli Pizzi et al., 2013). However, in this present study, we found that MPTP administration significantly elevated NAA in the striatum in induction PD day 1 and 7 mice, as well as in the SN in induction PD day 1 mice. Previously, we showed (Gao et al., 2013) that 6-OHDA rats showed a trend for increased NAA in the striatum. This present study went a step further and showed that NAA returned to normal levels in the striatum in induction PD day 21 mice by assessing metabolite changes at different time points during PD progression. The recovery of NAA was consistent with early studies in both people with PD and PD model animals (Lewis et al., 2012; Groger et al., 2013; Zhou et al., 2014). It is possible that temporary increase in NAA levels may be a consequence of the stress response to the drug or, more specifically, might compensate for the loss of dopaminergic neurons.

Glu is the major excitatory neurotransmitter in the mammalian central nervous system (CNS), which can be functionally invoked in virtually all activities of the nervous system (Lewerenz and Maher, 2015). In our present study, Glu levels in the striatum of induction PD day 1, 7, and 21 mice and the SN of induction PD day 1 mice were significantly increased compared with control rats. Similarly, our previous study also reported increased synthesis of Glu in the striatum of 6-OHDA rats (Gao et al., 2013). Both higher or lower levels of Glu can be noxious, as excessive Glu can stimulate activation of the N-methyl-D-aspartic acid (NMDA) receptor and induce excitatory toxicity (Grohm et al., 2012). In neurons, acute exposure to glutamate causes Parkin translocation to mitochondria in a calcium- and N-methyl-D-aspartate (NMDA) receptor-dependent manner, which disrupts some dynamic properties of mitochondria (Van Laar et al., 2015). Many studies in people with PD and animal models of PD have shown that mitochondrial dysfunction might be a defect that occurs early in the pathogenesis of PD (Subramaniam and Chesselet, 2013). Thus, we assume that the excitatory toxicity of Glu accumulation impairs mitochondrial function, and represents one of the potential metabolic mechanisms of PD.

GGC is one of the most important pathways involved in the metabolic coupling of astrocytes and neurons (Gallo and Ghiani, 2000). Changes in astrocyte-mediated GGC have been reported to be involved in the etiology of several neurodegenerative diseases, including epilepsy (Barker-Haliski and White, 2015), amyotrophic lateral sclerosis (ALS) (Sako et al., 2016), Alzheimer's disease (AD) (Chen et al., 2016), and Huntington's disease (HD) (Buren et al., 2016). Our present findings showed that levels of Gln significantly increased in induction PD day 1 mice, while no significant change in Gln levels were observed in induction PD day 7 and 21 mice in the striatum or SN. Meanwhile, the cycle-related enzymes (i.e., both GLS and GS) in the subcortex were significantly increased. We, therefore, hypothesize that this phenomenon could serve as a temporary strategy to protect neurons from Glu excitotoxic injury after the injection of MPTP when the balance of the GGC was rapidly compromised, as Glu levels only increased in the striatum. Considering previous studies and our current measurements of metabolites and enzymes, we speculate that the GGC is involved in metabolism mechanisms related to PD, and could be a potential target in the treatment or prevention of neuron loss and/or motor function impairment in PD.

Myoinositol (MI), one of most abundant metabolites in the human brain, acts to maintain glial cell volumes as an osmolyte. Additionally, activated glia with enlarged cell volumes tends to have an elevated mI (Chang et al., 2013). Although there was no significant change in mI between the PD and control group mice, we found the normalized mI value was always slightly higher in the striatum of MPTP-induced PD mice, especially in induction PD day 21 mice (*p* = 0.067). This finding suggested that glia may be activated at later stages in the MPTP-induced Parkinsonism model, which is in accord with the opinion of Niranjan (2014) that a glial reaction participates in the cascade of events leading to neuronal degeneration in PD.

Tau is considered the major organic osmolyte, in addition to mI, that can regulate brain osmotic adaption (Lien et al., 1991). Our study showed that Tau is significantly elevated in the striatum and SN by MPTP in induction PD day 1 and 21 mice. Additionally, the changes in Tau and mI indicated that osmolarity disorder occurs in the brain of PD mice. Furthermore, Tau was thought to have antioxidant properties and be able to reduce oxidative stress (Di Leo et al., 2004). These changes may represent a defense mechanism whereby increased Tau protects against the toxicity of MPTP, which can destroy the function of mitochondria and result in oxidative damage in the pathogenesis of PD.

Our study had several potential limitations for obtaining insights into the dynamic pathological mechanism leading to the development of PD. Indeed, people with PD at different severity levels would be the ideal subjects. However, the differentiation of these patients is not usually clinically feasible. In this present study, the MPTP mice model was used as an alternative to investigate changes in the metabolites underlying PD in a longitudinal study, from the initial to the final stages, which made it easy to control for the uniform state of disease. In addition, this present study adopted *in vitro* ^1^H NMR for metabolomics analyses of mice brain, as it allowed for straightforward comparisons with the *in vivo* MRS results. Further work will be required to confirm whether these dynamic alterations of metabolism occur in the brains of people with PD, from the proximal to the distal stages, using *in vivo* MRS.

In conclusion, our results demonstrate that the neuron loss and motor function impairment related with PD might be linked to an overactive GGC and altered membrane metabolism. The increased Glu, accompanied by an overactive GGC, can induce excitatory toxicity and impaired mitochondrial function in the brain, which may be strongly associated with the pathogenesis of PD. The change in NAA was more likely to have occurred to compensate for the loss of dopaminergic neurons. In addition, altered osmolality and activated glia may also be involved in the mechanism driving the onset of PD, as reflected by the changes in mI and Tau. Notably, more evidence is needed to identify the exact mechanisms involved that lead to the observed changes in Glu, Gln, and GABA concentrations, and to test those metabolites as novel therapeutic targets to arrest or ameliorate PD progression.

## Materials and methods

### Animals

Male C57BL/6 mice (the SLAC Laboratory Animal Co. Ltd. Shanghai, China), weighing between 20 and 26 g, were housed 5 per cage under the standard laboratory conditions (controlled temperature/humidity condition, a normal 12/12-h light/dark schedule with the lights on at 08:00 a.m.). They were given free access to standard chow and water during whole experimental process. All animal handling and surgery were performed in accordance with standard animal protection guidelines and were approved by the Institutional Animal Care and Use Committee of Wenzhou Medical College (wydw2016-0128).

### Preparation and treatment

After adapting to the environment for a week, 60 mice were randomly divided into control and PD groups. Mice in PD groups were injected intraperitoneally (i.p.) 30 mg/kg MPTP-HCl (M0896, Sigma-Aldrich) daily for 5 consecutive days (Jackson-Lewis and Przedborski, 2007), while the mice in control group received equal volume of normal saline. At 1, 7, and 21 days after the last injection, the mice were sacrificed and samples were harvested immediately. All brain tissues were frozen using liquid nitrogen and stored at −80°C until analysis.

### Behavioral test

Open-field test was performed to evaluate locomotor activity when mice were exposed to the novel environment. Standard protocol was conducted in a dark sound-attenuating apparatus to avoid outside interference. Activity was limited in the test chamber (25 × 25 × 45 cm) with white smooth floor divided by black lines into 16 equal squares 4 × 4. Behavior test was carried out between 19:00 and 21:00. Mice from control 21 day and induction PD day 21 groups were gently placed in the center of the chamber and allowed to move freely for 5 min. The total distance traveled, number of activity, activity time and immobility time were analyzed using an automated video tracking system DigBehv Animal Behavior Video-tracking System (Shanghai Jiliang Software Technology Co. Ltd. China) (Jing et al., 2011).

### Immunohistochemical staining

Three mice of subgroup were processed for immunohistochemical studies. The brains were carefully removed and serial coronal sections (5 μm) of striatum and substantia nigra (SN) were mounted on slides. The stained was handled in accordance with the instruction of immunohistochemical Kit (KIT-9710, Fuzhou Maixin Biotech. Co. Ltd. Fujian, China). Primary antibodies were: Tyrosine Hydroxylase (TH, as marker of DA terminals, 1:750, ab112, abcam), Glutaminase (GS, 1:50, ab156876, abcam), and Glutamine synthetase (GLS, 1:50, SCB). Images were obtained and analyzed using a fluorescence microscope (Nikon, Tokyo, Japan). Control sections in which primary antibodies or secondary antibodies were omitted showed no labeled cells.

### Sample preparation

Mice were sacrificed by decapitation, specimens of mesencephalon and bilateral striatum were dissected rapidly, snap-frozen in liquid nitrogen, and stored at −80°C until analysis. The metabolite extraction was referred to our previous method (ref). The frozen tissues were weighed into an Eppendorf tube. Following the homogenization by electric homogenizer with ice-cold methanol (4.0 mL/g) and ultrapure water (0.85 mL/g; (Beckonert et al., 2007)), the mixture was homogenized again with 2 mL/g of chloroform and 0.85 mL/g of ultrapure water using a vortex mixer, placed on ice for 15 min, and centrifuged at 10,000 g for 15 min at 4°C. Finally, the supernatant was carefully transferred into a new Eppendorf tube, lyophilized for 24 h, and stored at −80°C until NMR analysis.

### NMR spectroscopy

The lyophilized extract was redissolved in 0.5 mL of D_2_O containing 0.2 mM of TSP for NMR spectroscopy. D_2_O provided a field-frequency lock, and TSP was used as the chemical shift reference. ^1^H NMR spectra was acquired on a Bruker AVANCE III 600 MHz NMR spectrometer with a 5-mm TXI probe (Bruker BioSpin, Rheinstetten, Germany) at 298 K. The one-dimensional 1H NMR spectra of right striatum and SN were acquired using a standard single-pulse sequence with water signal presaturation (ZGPR). The acquisition time was 2.65 s per scan, and an additional 6 s relaxation delay was used to ensure full relaxation 128 transients were collected into 64 K data points with a spectral width of 12,000 Hz.

### Data processing of NMR spectra and multivariate pattern recognition

In all NMR spectra, the phase and baseline were corrected manually and referenced to the chemical shift of the methyl peak of lactate (CH_3_, 1.33 ppm) using Topspin (v2.1 pl4, Bruker Biospin, Germany). The spectra (5.90–4.50 ppm) containing the residual peak from the suppressed water resonance was set to the zero integral in the analysis. The remaining spectral segments were normalized to the total sum of the spectral intensity to partially compensate for differences in concentration of the many metabolites in the samples. Then spectra (0.5–10.0 ppm) were data-reduced to 1,100 integrated regions of 0.01 ppm width for multivariate pattern recognition analysis by SIMCA-P+ 12.0 software (Umetrics, Umea, Sweden). The supervised projection to latent structure discriminant analysis, Partial least squares-discriminant analysis (PLS-DA), was carried out for classifying the samples according to their common spectral characteristics as described previously (Liu et al., 2013). And another data-reduced to 7,334 integrated regions of 0.0015 ppm width corresponding to the region of δ 10 to 0.5 for quantitative analysis.

### Statistical analysis

Metabolite intensities relative to the sum of the total spectral integral among groups were calculated. Repeated measures ANOVA were used to compare the data from control and PD mice at different time points. For statistical comparison between two groups, Independent sample *t*-test was used. Statistical analyses were performed using SPSS (version 22, IBM, USA). The normality was assessed by the Kolmogorov–Smirnov test. Data are expressed as mean ± SE, and a significance level of 0.05 was used.

## Author contributions

YL and XZ data collection, statistical analyses and the initial draft of the manuscript. LP, KL and GB performed the experiments and reviewed the manuscript. LZ, CY, and CL contributed to the data discussion, and edited the manuscript. HG and ZY are the guarantors of this work and, as such, had full access to all the data in the study and takes responsibility for the integrity of the data and the accuracy of the data analysis.

### Conflict of interest statement

The authors declare that the research was conducted in the absence of any commercial or financial relationships that could be construed as a potential conflict of interest.
